# Multiple giant pilomatricomas of the scrotal: A rare case report from Syria

**DOI:** 10.1016/j.heliyon.2023.e19157

**Published:** 2023-08-22

**Authors:** Houda Alassil, Abdullah Omar, Samer Aldarf, Omar Alsamarrai

**Affiliations:** a Department of Dermatology and Venerology, Damascus Hospital, Syria; b Faculty of Medicine Syrian Private University, Damascus, Syria; c Department of Neurology Damascus University, Damascus, Syria

**Keywords:** Giant, Pilomatrixoma, Scrotum, Calcifying epithelioma, Malherbe

## Abstract

Pilomatrixoma, also known as calcifying epithelioma of Malherbe, is a cutaneous tumor originating from the hair matrix that commonly affects children. Pilomatrixoma is usually solitary and most commonly found on the face, neck, and upper torso, while the scrotum is considered a very rare site. We report a rare location and manifestation of pilomatrixoma as multiple, large, firm, calcified scrotal masses in a 32-years old man. An excisional biopsy was performed, and the diagnosis was confirmed.

## 1 Introduction

*Pilomatrixoma* is a rare, benign, slow-growing cutaneous tumor originating in the hair follicle matrix. Malherbe first described it in 1880. That is why sometimes it is called the calcifying epithelioma of Malherbe [1].

It exhibits a bimodal distribution with the highest incidence in children and adults over 50. The most common affected regions are the face (periorbital and preauricular), neck, and upper trunk, while the scrotum is considered a very rare site [2].

Pilomatrixoma is usually solitary. However, multiple forms have been reported in specific syndromes (such as Gardner syndrome, myotonic dystrophy, sarcoidosis, Steinert's disease, and Turner's syndrome) [3].

Clinically, it resembles common skin masses like sebaceous, epidermoid, or dermoid cysts. Due to this, it has often been misdiagnosed in 45–75% of cases [4].

The biopsy is the main mean of diagnosis, and surgical removal is the only treatment [4].

Here we report a rare case of giant scrotal pilomatrixoma presenting as multiple nodules in male patient.

## 2 Case presentation

A 32-year-old male patient presented to the dermatology outpatient department with a chief complaint of multiple masses over the scrotum. The onset of these lesions was not apparent because the patient has mental retardation and speech difficulties.

The urologists first noticed it as the patient was scheduled for varicocele surgery. After that, the patient was referred to the dermatology clinic due to the unusual appearance of these masses.

In the past medical history, the patient had scrotal mass excision 15 years ago without any histopathological report. No clear family history due to the mental and behavioral condition. Medical history only anti-psychotic had been declared.

He was not complaining of any associated pain or fever. Furthermore, no loss of weight or appetite was noticed during the past period.

**Fig. 1:**
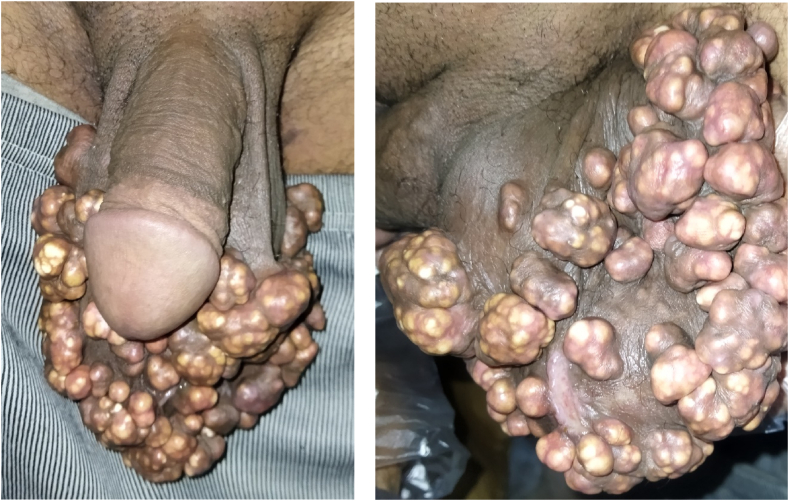
Shows the scrotum appearance with the lesions.

A large, firm, calcified, non-tender, skin-colored multiple nodules with irregular margins and lobulated appearance were palpitated by examination. These lesions were spread all over the scrotum. Fig. 1 shows the scrotum appearance with the lesions.

The lesions ranged from 0.5 to 2.5 cm in diameter as a solitary lesions. When The lesions gathered in large lumps, the most extensive measuring was 5.5 cm in diameter.

We could feel the testes separately, and there was no associated regional lymphadenopathy.

At first, a diagnosis of a scrotal sebaceous cyst was suspected.

The following deferential diagnosis was adopted: idiopathic scrotal calcinosis, subcutaneous cavernous lymphangioma, and pilomatrixoma.

That is why we ordered an excisional biopsy. The histopathological examination of the biopsy revealed the presence of nests of basaloid cells and mummified shadow cells with areas of calcification amidst shadow cells, which is consistent with pilomatrixoma.

**Fig. 2:**
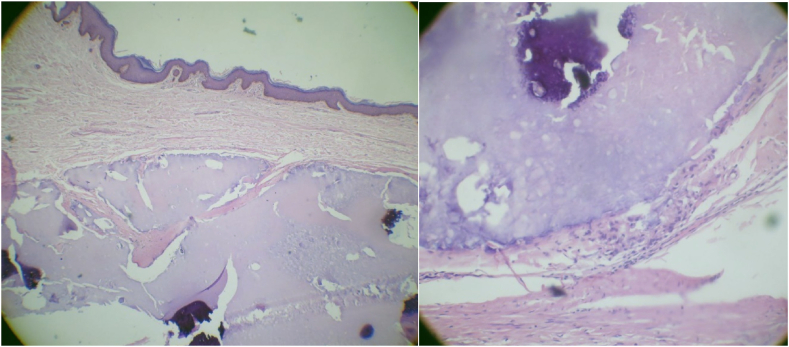
Shows histopathologic features.

No presence of infiltration to the adjacent tissues. Due to the advanced condition, more shadow cells were shown than basaloid cells. Fig. 2.

Patient's family had finical issue that prevent them to do extra genetic tests, beside the patient is ignored by them due to the lack of awareness and education level.

The patient was posted later for surgery, and all nodules lesions were excised. The histopathologic examination was done after the surgical removal of the lesions. And the result was identical to biopsy.

**Fig. 3:**
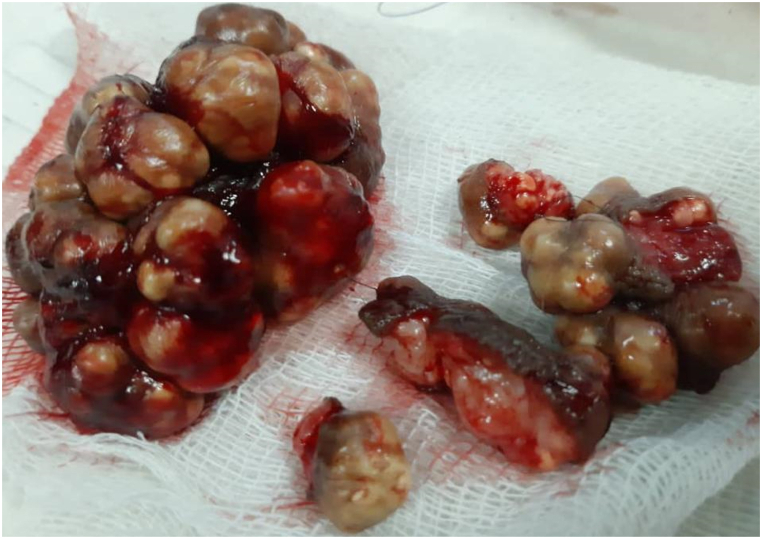
The lesions after excision.

The surgery was performed in several stages at the Urology Department to excise the lesions. Below is a picture of the lesions after excision. Fig. 3.

The patient was discharged without any significant complications. The patient was asked to visit the dermatology clinic to assess the condition's development. The patient did not comply with the instructions or review despite attempts to contact him.

## 3 Discussion

Pilomatrixoma, as a benign skin tumor, usually manifests as a single, nodular painless mass [2].

The scrotum is a rare location for pilomatrixoma emersion. The uncommon location gave our case its favor and made a distinction [4].

In addition to the rare location, the pilomatrixoma is usually single, while in our case, it is multiple, and the masses are variant in size. Also, the preferable age is childhood, and females are twice as likely as males to have a pilomatrixoma [4]. in our case, the lesion is affecting a 32 years old man.

Only a few research papers were published in the medical literature dealing with the presence of pilomatrixoma on the scrotum, so a comparison was made with what was published previously, and the comparison was as follows.

A case reported by Adhikari G and Jadhav GS [5] presented a giant pilomatrixoma. The lesion manifests as a large, firm, non-tender, pear-shaped sessile mass with irregular margins and a lobulated appearance.

A case reported by Malgras et al. [2] showed that the examination revealed a superficial, nodular poly lobed indurated tumor, not attached to the deep plane, 10 cm long axis.

The clinical manifestations cant differentiate between malignant and benign pilomatrixoma; thus, we depend on the histologic picture.

Regarding histopathology examination, Flynn A et al. [6] declare a report of two malignant pilomatrixoma; the histology shows moderate to marked pleomorphism nuclear of basaloid cells, atypical mitoses, central keratotic material, and infiltration of adjacent tissues by tumor cells. These features are not exist in our case.

## 4 Conclusion

Pilomatrixoma is a benign skin lesion, usually a single and non-tender mass. It is raising from the hair matrix, mostly affecting the pediatric population.

The case represented a rare deposition of pilomatrixoma in the scrotal skin. In addition to that, the lesion was giant, and the masses were multiple contrary to the usual.

## Author contribution statement

All authors listed have significantly contributed to the investigation, development and writing of this article.

## Data availability statement

Data will be made available on request.

## Additional information

No additional information is available for this paper.

## Funding statement

No funding resources.

## Ethical approval

Ethical approval was also taken from the faculty of medicine at Damascus hospital. According to Declaration of Helsinki.

## Consent for publication

Written informed consent was obtained from the patient for publication of this case report and any accompanying images.

## Declaration of competing interest

The authors declare that they have no known competing financial interests or personal relationships that could have appeared to influence the work reported in this paper.
